# Socio-sexual norms and young people’s sexual health in urban Bangladesh, India, Nepal and Pakistan: A qualitative scoping review

**DOI:** 10.1371/journal.pgph.0002179

**Published:** 2024-02-20

**Authors:** Prima Alam, Leesa Lin, Nandan Thakkar, Abhi Thaker, Cicely Marston

**Affiliations:** 1 Faculty of Public Health and Policy, Department of Public Health, Environments and Society, London School of Hygiene and Tropical Medicine, London, United Kingdom; 2 Laboratory of Data Discovery for Health (D24H), Hong Kong Science Park, Hong Kong, SAR, China; 3 Faculty of Epidemiology and Population Health, Department of Infectious Disease Epidemiology, London School of Hygiene and Tropical Medicine, London, United Kingdom; 4 School of Medicine, University of North Carolina, North Carolina, United States of America; 5 Indian Institute of Public Health Gandhinagar, Gandhinagar, Gujarat, India; University of Ghana, GHANA

## Abstract

In South Asia, young people face myriad challenges and opportunities regarding their sexual lives relating to varied experiences of norms and restrictions; gender norms and socio-sexual taboos limit communication around sexual health which in turn can affect sexual health outcomes. In this article we focus on norms affecting young people’s sexual health experiences in urban settings in Bangladesh, India, Nepal, and Pakistan. We conducted a scoping review of peer reviewed empirical studies based on qualitative data pertaining to young people’s experiences of sexuality and sexual health in Bangladesh, India, Nepal, and Pakistan. We searched four electronic databases for articles published (2010–2022), using terms relating to sexual health, young people, and South Asia. Sixteen articles met the inclusion criteria with sample size ranging from 9 to 180. The authors followed the Preferred Reporting Items for Systematic Reviews and Meta-Analyses extension for Scoping Reviews (PRISMA-ScR) guidelines for the design and analysis of this study. We synthesised the included articles using thematic analysis. The studies covered topics such as sexual health services and contraceptive use; sexuality education and communication; and gender and sexual violence. Recurring findings included: parental and societal expectations around premarital ‘sexual purity’ through abstinence; limited communication around sexuality between young people and parents/adults; gender norms limiting young women’s sexual and reproductive decision making; and an absence of research on experiences of sexual and gender minorities. We identified common themes as well as prominent gaps which must be addressed if we are to capture diverse experiences and build a better evidence base to improve sexual health services for young people in the region. The body of research fails to include experiences of young people with diverse gender, sexual orientation, and sex characteristics.

## Introduction

Accounting for almost a quarter of the global population, there are now more young people, aged 15–24 years, in the world than ever before [1,2]. This large and still-growing demographic has specific sexual health needs which must be adequately addressed to ensure a healthy transition to adulthood [3–6]. According to the World Health Organization [7], young people’s sexual health is grounded in their right to freely express their sexuality in consensual relationships, participate in activities such as marriage and having children, obtain accurate information about sexual issues, and access high quality sexual healthcare. The majority of the world’s 1.2 billion young people reside in low- and middle-income countries (LMIC) [1,3,4] where poverty and resource constraints limit access to sexual health services and education for many [1,4].

In South Asia, widespread seclusion norms and taboos around sexuality may undermine young people’s ability to make informed sexual and reproductive health (SRH) decisions [8–10]. Yet there is limited in-depth research about young people’s lived experiences of SRH in the region. Rapid urbanization as well rising internet use in recent years has also been influencing the ways in which young people experience and understand their own sexuality [1,11,12].

Young people in South Asia often have a poor understanding of sexuality and wellbeing–such as a lack of awareness of contraceptive use and sexually transmitted infections–partly because of limited access to SRH services and reliable information [1,13–17]. The situation is exacerbated by taboos relating to gender and sexuality which limit SRH communication between young people and adults–for example in Bangladesh and India [8,13–16]. In some countries such as Pakistan, young people seen to be deviating from socio-sexual norms of gender binaries and heterosexuality may face victimization and bullying as well as negative attitudes from their peers and family [4,18–20]. According to van Reeuwijk and Nahar, many young people approach adulthood with misconceptions and insecurities because of incomplete and incorrect information on sexuality which further perpetuates inequitable gender norms [8].

Recent research illustrates the challenges young people in South Asia face while accessing sexual health information. For example, a cross-sectional study performed in an urban district of Pakistan demonstrated that 62% of adolescents reported an inability to exercise their sexual and reproductive health and rights [21]. Roughly half (52%) of these adolescents highlighted structural barriers, citing a lack of conducive environments and limited literature among other constraints [21]. A 2019 study in Tamil Nadu, India demonstrated that young people hold several misconceptions regarding sexual health [22]. The authors noted a lack of comprehensive, formal sex education in schools, which may have prompted young people to seek alternative, less accurate sources of information, including peer groups, books, magazines, and videos [22]. Furthermore, in Bangladesh, a 2020 cross-sectional study showed that only 30% of older adolescent girls had appropriate knowledge on whether birth control impacts the sexual relationship of a couple [23]. Only about half (54.8%) of the same cohort had full information on sexually transmitted infection (STI) transmission [23].

As a consequence of these, and other, factors, many young people in South Asia may face negative sexual health outcomes such as STIs, unintended pregnancies, gender-based violence and risks associated with early marriage, and these may affect them more than the adult population [1,13–15,24,25]. While quantitative research has tracked the prevalence of these outcomes, in-depth qualitative research may help to describe, and find reasons for, young people’s sexual behaviour and its social context [26].

According to the World Health Organization, sexual health encompasses an individual’s physical, emotional, mental, and social wellbeing with regard to sexuality [7]. Lived experiences of these aspects of sexuality are embedded within a broader sociocultural context that influences young people’s vulnerability or resilience to adverse sexual health outcomes [27,28]. For example, consensual same-sex sexual behaviour may have different consequences depending on varying social structures, such as prevailing norms and laws around compulsory heterosexuality [27].

Socio-demographic changes may also be shaping young people’s experiences of their sexuality [28]. Rapid urbanization is also leading to increasing marginalization of urban poor youth populations as well as rising urban health disparities within LMIC settings [28–30]. The urban population of South Asia is projected to rise to over 880 million by 2030 from its current 632 million, with the highest annual growth rates of change in Nepal and Bangladesh [31]. Having up-to-date information on qualitative research around young people’s experiences is important to consolidate our current understandings of sexuality across these developing landscapes.

The huge size of the population of young people, the increasing urbanization of the populations in South Asia and the need for good SRH services suggest that it is crucially important to understand young people’s sexuality to inform services and ensure the best outcomes for this large and expanding group. We searched two leading research databases (Cochrane Library and Web of Science), using the terms “sexuality”, “young people”, “South Asia” and synonyms, and found that no relevant review of the literature on young people’s sexuality in South Asia has yet been undertaken.

Here we present our review of qualitative research on young people’s experiences of sexuality in urban settings of four South Asian countries–Bangladesh, India, Pakistan, and Nepal–published within the past decade (2010–2022). As well as sharing historical cultural ties, the selected countries have some of the highest percentages of youth population in the region [32,33]. Additionally, all four countries have undergone similar sociodemographic and economic transitions in recent years as well as trends in SRH outcomes [34–36]. For the purpose of this review, we defined “young people” as individuals aged 15 to 24 years [33]. We examine what the literature can tell us about prevalent sexuality norms and restrictions, and how these affect young people’s lives. We also identify major gaps in the research. This review is part of the first author’s doctoral research which explored meanings and perceptions of sexuality through lived experiences of young people in Dhaka, Bangladesh [37–39].

## Materials and methods

### Search strategy

We searched for all studies reporting qualitative data on young people’s sexuality from Bangladesh, Nepal, India, and Pakistan. We searched four databases (EMBASE, Global Health, Ovid-MEDLINE, and PsycINFO) on 16 January 2021 and limited year of publication from 2010 to 2021 to review contemporary sexual health issues. The authors also ran an updated search on 30 December 2022 to incorporate peer-reviewed studies published since the previous search (16 January 2021) till present. Our search strategy (S1 Table) was based on the on the ‘Population Interest Context’ (PICo) method. We combined MeSH terms and key words relating to young people (e.g. youth, young people, and young adult*), SRH (e.g. sexua*, sexual health, and reproduc*), South Asia (e.g. Bangladesh, India, Pakistan, Nepal, and Sout* Asia) and research type (e.g. qualitative, ethnograph*, experience*, focus group*, and interview*). Additionally, we searched citations of all articles identified as relevant. We referred to the Preferred Reporting Items for Systematic Reviews and Meta-Analyses extension for Scoping Review (PRISMA-ScR) guidelines for design, analysis, and interpretation of results (S1 PRISMA 2020 checklist) [40]. The authors also referred to the 21-item Enhancing transparency in reporting the synthesis of qualitative research (ENTREQ) statement to facilitate reporting [41].

### Inclusion criteria

We included all peer-reviewed qualitative or mixed methods empirical studies on young people’s experiences of sexuality/sexual health based in Bangladesh, India, Pakistan and Nepal, and published between 2010 and 2022. We excluded materials that were not published in peer-reviewed journals or did not incorporate empirical evidence (e.g. book chapters, review articles, theses). Articles with a study population that did not include any young people aged 15 to 24 as defined by the United Nations Population Fund (i.e. articles with only participants under the age of 15 or over the age of 24) were excluded. Articles where the age of participants was indistinguishable (i.e. age not reported or without disaggregated data for adult population) were also not included. Only studies which encompassed urban respondents–either exclusively or in addition to rural respondents–were included, and studies focussed solely on rural areas were excluded (S2 Table).

### Data extraction and analysis

Two reviewers independently screened titles, abstracts, and full text for inclusion and extracted data from relevant articles. Before data extraction, a coding framework (S1 Text) was developed centring on study characteristics, key findings and recommendations, prominent norms, and lifeworld domains emerging from the articles. Two authors independently coded full-text articles and compared the results. A third reviewer assessed the coding in case of any discrepancies. The findings were organized and interpreted thematically through a phenomenological lifeworld perspective by identifying and synthesising most prominent and recurring socio-sexual norms to emerge from the included articles [42,43]. This involved coding each article according to the coding framework and then synthesizing themes across all included articles.

### Quality appraisal

Two authors appraised all 16 articles using the Critical Appraisal Skills Programme (CASP) Qualitative Checklist (S3 Table). The third reviewer assessed the appraisal scores for any inconsistencies. We considered all the articles to be of high quality as all articles fulfilled criteria of reporting study validity, clarity of results, and value/contribution of research. As such, none of the eligible articles were excluded after quality appraisal.

## Results

### Search outcome

**Fig 1:**
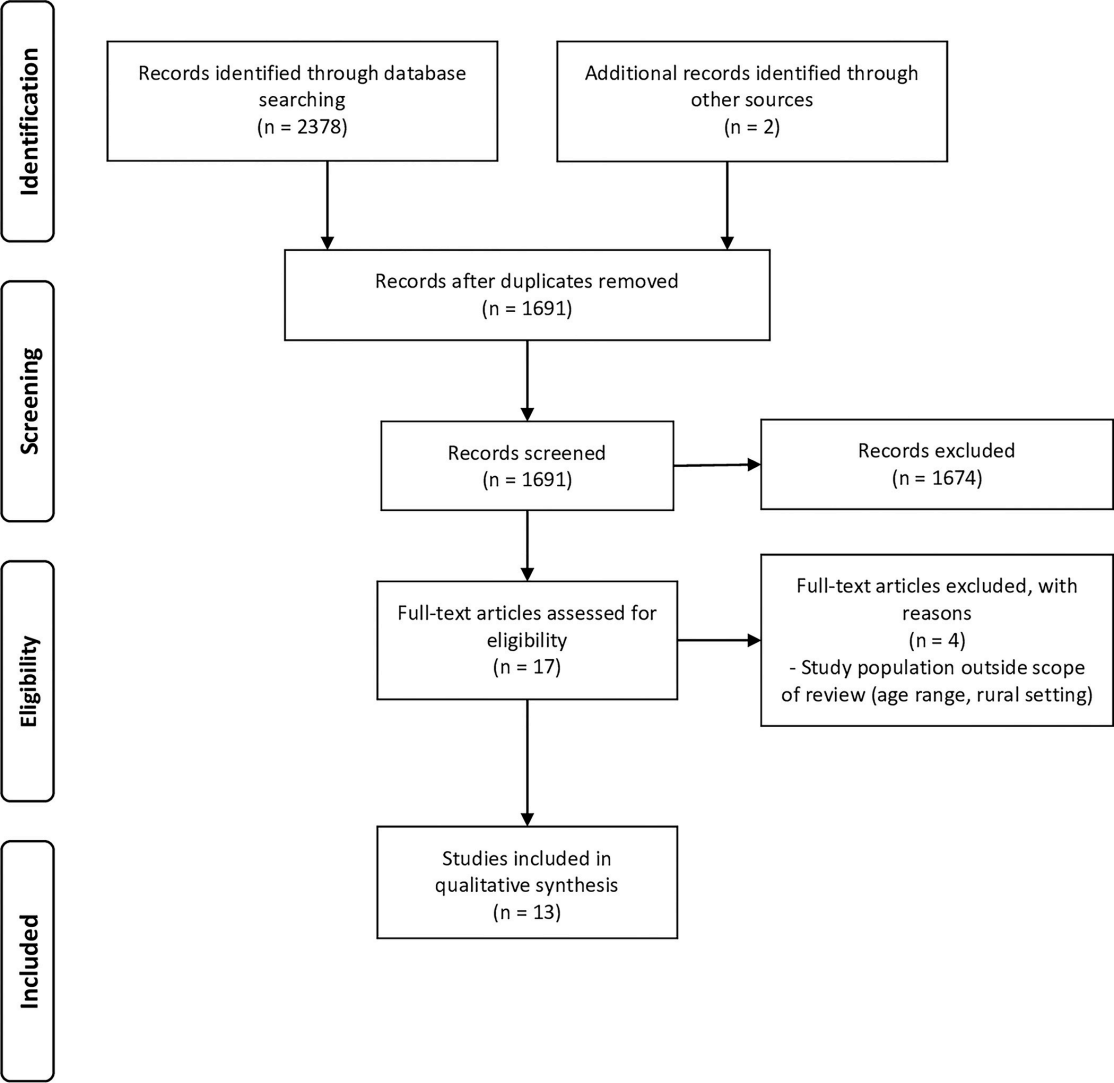
Flow diagram of study selection process.

Of 2962 articles identified from four databases using different keywords, 1008 were duplicates and 1916 proved not to be relevant (i.e. not related to young people’s sexuality in South Asia) after title and abstract screening. Related citations and reference lists of all relevant articles were also checked, and two further articles were retrieved. We assessed 40 articles for eligibility and 24 of these were excluded after full-text screening as the study population was outside the scope of this review (i.e. the study solely utilized quantitative methods, respondents were not aged 15 to 24 years, or only rural study populations were considered). Finally, 16 articles were included for data extraction and thematic analysis (Fig 1).

### Study characteristics

Five of the sixteen included articles were studies conducted in Nepal which focussed on barriers to sexual health services, prenatal intimate partner violence (IPV) and attitudes towards dating. Five articles were based on research in India and included data around sexual health needs, heterosocial friendship dynamics at school, understandings of gender and sexual violence, and an intervention contesting restrictive mobility norms. Four studies were based in Karachi and Islamabad in Pakistan and explored perceptions of contraceptive use and young women’s experiences of marriage preparation. The remaining two articles were based on experiences of sexuality education and communication between parents and youth in Bangladesh. Seven of the included studies were funded by European institutes or organizations, one was funded by an Indian institute, one by a Japanese organization, and another by an American organization. The remaining six studies either did not specify donors or did not receive funding.

All included studies were cross sectional in design and only two articles–based on the same study in India–used both qualitative and quantitative methods. Interviews were carried out between 2006 to 2019 and the data collection period ranged from 10 days to a year. Two articles did not specify the year or duration of data collection and five articles did not report the exact length of data collection. Data collection methods for all studies included one-to-one in-depth interviews (IDIs) and/or focus group discussions (FGDs) with young people as well as key informant interviews. Apart from two studies which used online interviews [44,45], all interviews or FGDs were conducted face-to-face.

Eleven articles were based on data collected in urban settings and five included both rural and urban data collection. Five of the 11 articles exclusively in urban settings collected data from urban slums in Mumbai and Lucknow, India; Kathmandu, Nepal; and Karachi and Islamabad in Pakistan. Beyond study location, respondent characteristics also varied by education level, gender, age, marital status, socioeconomic status, religion, and ethnic group. For example, five articles recruited students from formal educational institutions (schools, colleges, and universities). Two articles–based on the same study in Nepal–focused on data from both rural and urban educational institutions as well as youth clubs.

In terms of study population, total sample size varied from 9 to 180 participants. Seven articles focussed on unmarried respondents, three on married or engaged participants, and five combined both married and unmarried/engaged young respondents. One article did not specify the marital status of respondents. Most articles included both men and women as participants while three focused on women and two on men. The socioeconomic status (SES) of study participants varied, with six articles looking at slum residents or young people from a lower caste and six articles including urban middle-class participants. Only two papers included participants from both middle and lower SES and the remaining three articles did not specify.

While included articles had different topics, the main research areas could be summarized as exploring sexual health services and contraceptive use (n = 6); sexuality education and communication (n = 5); gender norms and sexual violence (n = 5). Articles used the terms boys/girls and men/women and here we use the same language as the relevant article where possible. Table 1 summarizes the characteristics of the 16 included articles.

**Table 1:** Summary of study characteristics of 16 included articles.

Author, year	Country	Study design	Data collection		Study setting		Study population				Study topic
			Methods	Study duration	Setting	SES	Gender	Age	Marital status	Sample size	
Bankar et al., 2018 [46]	India	Cross sectional qualitative	In-depth interviews (IDIs)	Not reported	Urban slums (Mumbai)	Slum residents	Women	18–24	• Unmarried • Married	10	Contesting restrictive mobility norms
Bansal et al., 2021 [47]	India	Cross sectional qualitative	Focus group discussions (FGDs)	4 months	Urban slums (Lucknow)	Slum residents	• Boys • Girls	15–17	• Unmarried • Engaged	40	Adolescents’ experiences and societal norms regarding gender
Brahme et al., 2020 [48]	India	Cross sectional qualitative	FGDs	2 months	Urban colleges (Pune)	Mixed	• Boys • Girls	20.82 ^a^	Not reported	74	Sexual behaviour and needs (HIV/STIs)
Camellia et al., 2021 [49]	Bangladesh	Cross sectional qualitative ethnography	• FGDs • IDIs	1 year	Urban (Dhaka)	Middle class	• Boys • Girls	15–19	Unmarried	72	Communication on sexuality in the youth-parent relationship
Deuba et al., 2016 [50]	Nepal	Cross sectional qualitative	IDIs	5 months	Urban slums (Kathmandu)	Slum residents	Women	15–24	Married (pregnant)	20	Prepartum IPV
Farid-ul-Hasnain et al., 2013 [51]	Pakistan	Cross sectional qualitative	FGDs	1 month	Urban (Karachi)	Mixed	• Men • Women	17–21	Unmarried	42	Knowledge, attitudes, beliefs and perceptions on contraceptive use and HIV prevention
Gautam et al., 2016 [52]	Nepal	Cross sectional qualitative	IDIs	2 months	• Urban • Rural (Dang)	Hill Dalit community	• Men • Women	15–24	• Unmarried • Married	22	Barriers to using sexual health services
Hamid et al., 2010 [53]	Pakistan	Cross sectional qualitative	• FGDs • IDIs	Not reported	Urban slums (Islamabad)	Slum residents	Women	15–24	Engaged to be married	34	Experiences of marriage preparation; understanding of transition to marriage and childbearing
Iyer, 2018 [54]^b^	India	Mixed methods with ethnography	• FGDs • IDIs	5 months	Urban secondary schools (New Delhi)	Middle class	• Boys • Girls	15–17	Unmarried	41 (180) ^c^	Heterosocial dynamics within school peer cultures
Iyer, 2017 [55]^b^	India	Mixed methods with ethnography	• FGDs • IDIs	5 months	Urban secondary schools (New Delhi)	Middle class	• Boys • Girls	15–17	Unmarried	41 (180) ^c^	Understandings of gender and sexual violence
Khan & Raby, 2020 [44]	Bangladesh	Cross sectional qualitative	IDIs (virtual)	2 months	• Urban • Rural	Not reported	Men	19–24	Unmarried	9	Experiences of sex education
Nishtar et al., 2013 [56]	Pakistan	Cross sectional qualitative	FGDs	4 months	Urban slums (Karachi)	Slum residents	• Men • Women	18–24	Married (with ≥1 child)	50	Myths and fallacies about male contraceptive methods
Regmi et al., 2010 [57]	Nepal	Cross sectional qualitative	• FGDs • IDIs	Not reported	Urban and rural colleges/youth clubs (Kathmandu and Chitwan)	Not reported	• Boys • Girls	18–22	• Unmarried • Married	50	Barriers to using sexual health services and condom-use
Regmi et al., 2011 [58]	Nepal	Cross sectional qualitative	• FGDs • IDIs	Not reported	Urban and rural colleges/youth clubs (Kathmandu and Chitwan)	Middle class	• Boys • Girls	15–24	• Unmarried • Married	106	Attitudes towards dating and sex
Sekine et al., 2021 [59]	Nepal	Cross sectional qualitative	• IDIs • Key informant interviews	10 days	• Urban • Rural	Middle class	• Men • Women	15–24	• Unmarried • Married	24 (70) ^d^	Factors influencing contraceptive use and childbearing
Shivji et al., 2021 [45]	Pakistan	Cross sectional qualitative	IDIs (in-person and virtual)	Not reported	Urban (Karachi)	Mixed	Men	18–21	Unmarried	22	Young men’s puberty experiences

ESRC: Economic and Social Research Council; FGD: focus group discussion; ICMR: Indian Council of Medical Research; IDI: in-depth interview; IPV: intimate partner violence; SES: socioeconomic status; SIDA: Swedish International Development Cooperation Agency; STINT: Swedish Foundation for International Cooperation in Research and Higher Education.

^a^ Article reported mean age of respondents

^b^ Articles based on same study

^c^ N = 180 students completed initial questionnaire, of which 41 were interviewed individually or in focus groups

^d^ N = 70 of which 24 were 15–24 year old men and women.

### Prevailing sexuality norms in South Asia

**Fig 2:**
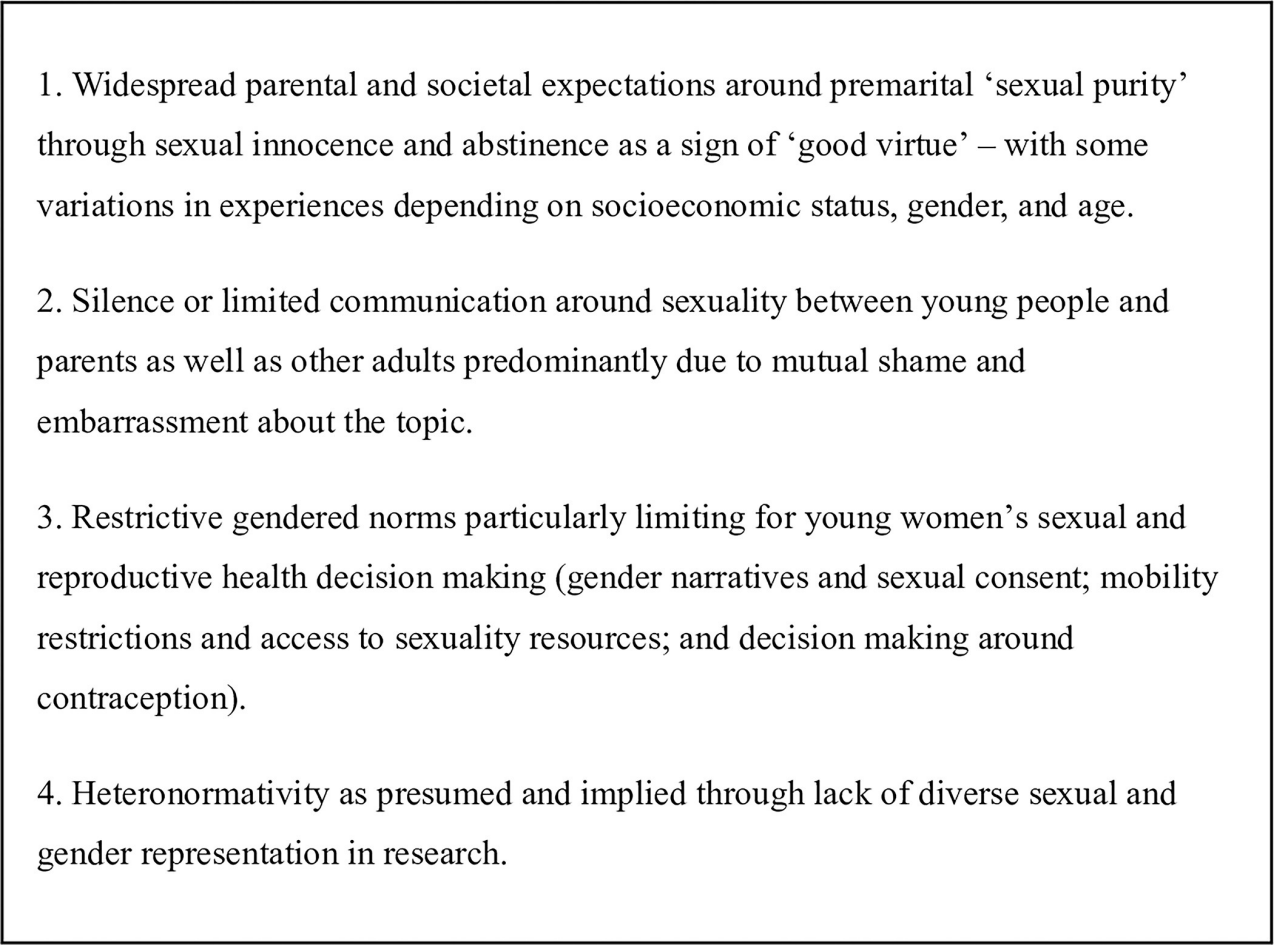
Themes around sexuality norms as reported in included articles.

Four interrelated themes of norms and restrictions around sexuality were widely reported across published articles, as shown in Fig 2.

Table 2 summarizes the prevailing norms as well as how young people are reported to be experiencing and navigating these. Corresponding sexual health consequences and recommendations as outlined by included articles are also presented in the table.

**Table 2:** Summary of sexual health consequences of, ways of navigating, and recommendations around socio-sexual norms as discussed in 16 included articles.

Author, year	Norms or constraints identified in included studies	Sexual health consequences of norms/constraints reported	How do young people navigate norms?	Recommendations of study
Bankar et al., 2018	**Gendered mobility restrictions** • Restrictive visibility norms for girls and women in public spaces **Premarital sexual purity** • Good virtue: modesty, respectfulness, proficiency in household chores and, above all, sexual purity	• Harmful gender norms act as structural barriers to many public health interventions • School dropout and early marriage has a negative effect on girls’ wellbeing • Gender discriminatory practices and social patterning of health inequalities	• Greater negotiation skills through intervention helped win parents’ trust in ability to be safe and ‘respectable’ in public • Parents became supporters thus co-producing daughters’ identity as doing ‘good work’	Not reported
Bansal et al., 2021	**Premarital sexual purity and silence on sexuality in youth-parent/adult relationships** • Stigma associated with adolescent relationships and its influence on individual norms • Lack of communication between adolescents and adults due to taboos around sex	- Experiences of shame and stigma from premarital relationships, particularly for girls - Health consequences of gender-based harassment	Not reported	• Gender-based harassment in public spaces and inadequacy of current systems demands national-level response • Boys can be allies in preventing harassment against girls • Encourage greater parent–child communication on relationship topics to foster gender-equitable attitudes
Brahme et al., 2020	**Silence on sexuality in youth-parent/adult relationships** • Cultural barriers and taboo to discuss sexuality; no open discussion with parents	• Sex education not adequate to bring maturity, leading to problems such as unwanted pregnancies, and unprotected sex	• Use alternative sources of knowledge on sexuality (e.g. peers, internet, pornography) - Google search sexual health information	• Parental involvement and open discussions on sexuality preferred by students • Use of innovative sexual health mobile app • Develop holistic approach for culturally-sensitive sex health education and life skills model
Camellia et al., 2021	**Silence on sexuality in youth-parent relationships** • Lack of communication on sexuality in youth-parent relationship due to socio-sexual norms • Taboo around cross-sex communication, particularly between mothers and sons **Premarital sexual purity** • Sexual abstinence to uphold family’s respectability	• Limited access to information about own bodies and physical changes during puberty; particularly problematic for boys	• Young people think it unnecessary and shameful to discuss sexual pleasure with parents • Silence used to protect good boy/girl image and be seen to respect parents’ values • Internet perceived as more effective source of information than parents	• In-depth understanding of silence when researching sexuality to avoid generalising silence as always oppressive • More knowledge about when, on which topics, or for whom internet is more effective than parental communication
Deuba et al., 2016	**Gendered decision-making around sex and childbirth** • Lack of sexual consent for pregnant young women • Son preference	• Experiences and health consequences of IPV (e.g. negative birth outcome, trauma) • Misconceptions around son preference (e.g. having sex during pregnancy helps to get a son)	• Tolerate and accept husbands’ abuse due to economic dependence • Seek informal support from close family members • In-laws interfere to prevent escalation of physical abuse	• Health workers can increase awareness in men and families with strong son preference • Interventions to improve pregnant women’s abilities to tackle mental health consequences • Involve community and in-laws in IPV prevention
Farid-ul-Hasnain et al., 2013	**Silence on sexuality in youth-adult/parent relationships** • Lack of intergenerational communication around sexual health	• Knowledge gap concerning HIV/AIDS and contraceptive use • Susceptibility to HIV, unwanted pregnancies, unsafe abortions and sexual abuse or violence • Fear of harassment and violence from elders	• Rely on media and peers for sexual health information	• Improve access to quality clinical services with effective treatments, and accurate sex education • Supportive adult guidance on SRH matters and educational and economic opportunities • Develop public health strategies and curriculum-based program targeting individual behaviours and socio-structural factors that act against safe sex
Gautam et al., 2018	**Silence on sexuality in youth-parent/adult relationships** • Young people uncomfortable discussing sexual health with elders, family, or service providers of opposite sex	• Low utilization of sexual health services	• Prefer visiting other health centres if service provider at nearest health facility is of opposite sex	• Community-based educational programs and accessible youth-friendly service centres to encourage use of services
Hamid et al., 2010	**Silence on sexuality in youth-adult/parent relationships** • Culture of silence around sexuality and transition into adulthood **Premarital sexual purity (submissiveness)** • Young women socialized into submissiveness and abide by rules to be “good daughters”	• Lack of proper understanding of how to use and access contraception • Women’s lack of control over future reproductive health	• TV as peer (e.g. contraceptive adverts) • Ask parent-approved information sources about sexuality and childbearing near time of marriage (e.g. older cousins, sisters, aunts) • Abide by rules and trust family support will ensure security in future life	• Create community-based informal groups for young women to discuss sexuality, childbearing, and other marriage-related issues • Uplift women’s self-identity and integrate women into decision-making with parents and husbands
Iyer, 2018	**Gender segregation/brother-sister relationships at school** • Institutional narratives of *rakhi* (brother-sister) relationships with boys respecting girls as sisters **Premarital sexual purity** • Sexual activity among students exception rather than norm reflecting middle-class norms of premarital purity	• Perpetuates regressive gendered power dynamics and restrictive concept of sexuality • Frames all male sexual desire as derogatory towards women and women who express sexual desire as unworthy of male respect	• Peer romance important source of sexual learning for students to explore experiences of pleasure and intimacy side-lined within institutional risk-based narratives of sexuality • Reject norms emphasising gender inequalities by forming heterosocial friendships	• Further research around gender dynamics within heterosocial friendships in India
Iyer, 2017	**Gender norms and sexual violence** • Institutionally reinforced gender norms through narratives of good boy/hero masculinity and can-do (high-achieving, independent femininity)/vulnerable girlhood	• Confusion around boys’ understandings of sexual violence and sexual pleasure • Frustration for girls as can-do girlhood often undermined and vulnerable femininity affirmed through heightened restrictions	• Aligning with narratives of ‘good boy’/‘hero’ masculinity and ‘can-do girl’/‘vulnerable girl’ • Challenging vulnerable narratives through can-do girlhood valued within girls’ peer cultures, as celebration of modern Indian woman	• exuality in more positive terms, in formal school settings or more informal interactions • Talk about issues around consent • Schools can better support young people as they learn about gender and sexuality from diverse and contradictory sources
Khan & Raby, 2020	**Silence on sexuality in youth-adult/parent relationships** • Dominant discourse that sexuality is private, shameful and adult matter reproduced at home and school **Premarital sexual purity** • Parents expect sexual abstinence until marriage **Gendered learnings around sex and sexuality** • Learnings around sexuality strongly gendered	• Gendered knowledge about sex and sexuality reproduces hegemonic masculinity, and undermines women’s consent • Uncertainty, isolation and vulnerability around sex and sexual health	• Accessing alternative sources of information through peers, pornography and learning by doing	• Good quality sex and sexuality education for Bangladeshi boys
Nishtar et al., 2013	**Gendered decision-making around contraception** - Men and mothers-in-law main decision makers in contraception use and childbearing	• Low use of male contraceptives due to cultural beliefs, and myths and fallacies (e.g. impotency associated with condom use) • Women have little control in adopting contraception	Not reported	• Set up user forum for advocacy about male contraception • Target cultural issues through peer counselling for husbands and mothers-in-law, and train family planning service providers • Use public messages and easy to understand booklets in local languages
Regmi et al., 2010	**Silence on sexuality in youth-adult and peer group relationships** • Embarrassment while talking about sexual health with friends of opposite sex, family, and even sexual partners	• Barriers to accessing information or services on sexual health • Poor negotiation and decision-making skills can lead to unsafe sex • Misinformation about sexuality from peers	• Avoid sexual health services • Engage in unsafe sexual practices • Seek sexual health advice from peers	• Ensure confidential and respectful services • Provide necessary negotiation skills to avoid risks of HIV/STIs and unwanted pregnancy • Free/discounted sexual health services, and peer education programs • Establish convenient youth-friendly service centres
Regmi et al., 2011	**Gendered decision making around sex** • Girls have less negotiating and decision-making power around sex **Premarital sexual purity**	• Girls perceived to face physical, mental, and social consequences of sexual relationships	• Learn about love, dating, and relationships from media • Chatting online in cyber cafes to find partners • Meet and form partnerships with opposite sex while at college or in their community	• Discuss dating practice in formal and informal education to promote safer sex
Sekine et al., 2021	**Gendered decision-making around sex and childbirth** • Patriarchal norms and power imbalances between spouses limited women’s family planning decision-making • Son preference • Limited mobility barrier to accessing family planning information	• Misconceptions and fear around contraceptive methods among men and women • Women have limited autonomy in decision making around family planning • Lack of access to family planning information for women	Not reported	• Need to challenge restrictive sociocultural norms by targeting families and communities so that adolescent girls become empowered to exercise greater control over contraceptive use
Shivji et al., 2021	**Silence on sexuality in youth-parent/adult relationships** • Challenging to communicate about experiences of puberty with others (e.g. elders) and information was difficult to access	• Young men unable to obtain reliable information on puberty • Negative psychological and emotional impact of puberty experiences (anxiety, embarrassment, and isolation)	• Coping strategies (e.g. strong social support through peers, improving ‘self-control’) to help deal with negative experiences	• Further research on development of health promotion programs for adolescent boys • Need for culturally appropriate puberty education and various facilitating factors (e.g. home education programs) that may improve the puberty experiences for future young men

#### Widespread expectations around premarital ‘sexual purity’ through sexual innocence and abstinence as a sign of ‘good virtue’

Seven articles explicitly described parental and societal disapproval of premarital sex as a common phenomenon experienced by most young people [44,46,47,49,53,55,58]. Young people across these studies reportedly perceived “sexual innocence” and premarital sexual abstinence as a way of signalling their “good virtue” to their parents and wider family members. While sexual abstinence before marriage was reported as a widespread expectation for young people, experiences of this norm varied by socioeconomic status, gender, and age.

Regarding socioeconomic status, in addition to sexual abstinence and not mixing with the opposite sex, middle class respectability required young people to focus on academic achievements, while young women living in slums were required to display “good virtue” through competence in household chores [44,46,47,49,53–55].

In the context of young women living in slums, two studies in India and Pakistan linked “sexual purity”–described as premarital sexual abstinence as well as “sexual innocence and ignorance” as a sign of virginity–very closely with being good daughters who take care of domestic duties [46,53]. For example, Bankar and colleagues identified modesty, respectfulness, proficiency in household chores, and “sexual purity” as traditional norms expected of young unmarried women. The authors explore how a sports-based program had given participants greater skills to negotiate restrictive mobility norms with parents [46]. In the absence of such interventions, a study based in Pakistan revealed young women engaged to be married had been “socialised into submissiveness” leading to them being more vulnerable to reproductive ill health in the future. The participants abided by rules to be “good daughters” as they trusted that continued family support would ensure security in future life [53].

A study of middle-class boys and girls in Dhaka, Bangladesh suggested that their parents saw sexual abstinence as a way of upholding the family’s respectability [49]. However, respondents in this study said they were able to navigate this norm by using silence around sexuality with their parents as a way of protecting good boy/girl image, be seen to respect parents’ values, and avoid mutual embarrassment [49].

Age was also a noteworthy factor in determining how young people perceived and experienced expectations around romance and sex. Iyer’s study with students aged 15–17 reported that sexual activity among participants appeared to be the exception rather than norm which could reflect “middle-class norms of premarital purity” as well as due to “the younger age range of participants in this study” [54]. Age-specific norms around romantic relationships were also explored in an article where participants of a Nepal-based study–aged 15–24 –believed the community viewed dating positively if practiced by “emerging adults” over the age of 18 rather than adolescents in their early teens [58].

Participants from the above study demonstrated a mostly positive attitude towards dating and sexual relationships. Young men and women shared their experiences of circumventing familial expectations of sexual abstinence by going out on dates rather than bringing partners home. The authors reported that unmarried young women challenged expectations of sexual abstinence and that considerations such as trust and opportunity were important in deciding whether to engage in sexual relations before marriage [58]. Likewise, a study based in India found that, despite social taboos against romantic interactions, adolescents were engaging in intimate relationships [47].

Additionally, Indian college students from another article reportedly believed that age and maturity–in terms of decision-making and responsibility–were also factors in deciding when to have sex for the first time [48]. The majority of respondents in Brahme et al.’s study in Pune, India said they believed that the “ideal age for initiation of sex” was between the age of 18 and 23 years as young people within this age range were better positioned to make responsible decisions compared to secondary school-aged youth [48].

#### Limited communication around sexuality between young people and parents due to embarrassment

In addition to premarital sexual abstinence, nine articles specified a culture of silence around sexuality as a widespread norm [44,45,48,49,51–53,57,59]. Overall, young people felt embarrassed discussing sexual health with parents, elders, health professionals and friends of the opposite sex, and even sexual partners [45,52,53,57,59]. As a 24-year-old man in Bangladesh explained: “[Due to socio-cultural norms] people feel shy if we talk directly on sex” [44]. In addition to embarrassment, Farid-ul-Hasnain and colleagues suggested that the lack of intergenerational communication around sexuality was due to young people fearing harassment and violence from elders as a consequence of expressing sexual curiosity [51].

While most young people were curious, discussing sexuality was a taboo as it went against expectations of “good virtue” and sexual abstinence before marriage. As Khan and Raby [44] observed, the dominant discourse that sexuality is a private, shameful, and adult matter appeared to be reproduced through silence around the subject at home and educational institutions across all four countries. When parents did offer limited guidance, “this was only to tell them not to have sex until marriage, and to forbid girls from mixing with boys” [44]. The authors explore this silence or limited guidance as a way of exercising disciplinary power over young people to control their sexual behaviour and reproduce discourses like sexual abstinence before marriage.

Although respondents across all nine articles mentioned feelings of embarrassment or shame, Indian college students in a 2020 study said that they still wanted active parental involvement and open discussions on sexuality [48]. Most students learned about sexual health from peers or internet searches but felt their parents were best positioned to provide reliable information. However, the article did not provide further details on which particular ‘sexual issues’ the participants wanted to discuss with parents [48]. Participants in Camellia et al.’s ethnographic study specified that they would have benefited from information around physical changes that occur during puberty but preferred not to talk about romance, love, or sex post-puberty because they felt awkward and uncomfortable. A 17-year-old focus group participant in the study explained: “The thought of speaking with parents about love or sex feels simply awkward. We are not even comfortable watching kissing scenes on television in the presence of our parents” [49].

Additionally, Camellia and colleagues [49] observed a temporal dimension of changes in youth-parent communication needs during and after puberty: as adolescents, participants wanted their parents to have open discussions about puberty but post-puberty they preferred to not communicate about sexual desire and dating. Likewise, a study of young men’s experiences of puberty in Karachi, Pakistan demonstrated that respondents wanted more guidance during this challenging period of transition but were unable to communicate this need to older relatives [45]. As one 21-year-old respondent said: “Our culture does not allow such communication with elders. That was the main barrier that I could not communicate regarding my puberty with elders” [45].

Despite this limited communication, young people were curious about their sexuality, and reiterated the need for sexual health education [44,45,48,49,51–53,57,58]. However, as with expectations of sexual abstinence, the ways young people navigated this limited guidance varied. For example, young women engaged to be married in Pakistan expressed curiosity about married life, but said they learned that talking about sexuality was a sign of having no shame as one 19-year-old respondent described [53]: “I am looking forward to my marriage and I want to ask questions but I do not talk about this with my mother…she doesn’t even know I menstruate. How can we talk about these things?”

Where possible, young people navigated this gap between curiosity and silence by looking for information around sexuality via the internet, media, pornography, and their peer groups. Five articles mentioned the importance of the internet/media as well as peers as alternative–and, most of the time, preferred–sources of sexual health information for young people [44,45,48,49,51,58]. Respondents with access to the internet found Google searches and YouTube to be a more effective resource than parents, as this 16-year-old student from Bangladesh explained: “In no way our parents can explain sex better than those videos. They will die out of shame” [49].

Young people were also routinely exposed to sexual content through the media without having to actively search for it. A 20-year-old woman from Nepal described how being inundated with such content affects young people [58]: “We watch TV and films, read papers and listen to the radio…It is all about sex. We become emotional and attempt to do such things.”

Access to, and reliability of, alternative resources varied across contexts. For example, young men in Bangladesh received information about sexuality from their peers but were of the view that peers were “unreliable sex educators” [44]. In another study, soon-to-be-married women of a slum in Pakistan had limited and vague information from contacts approved by respondents’ mothers, such as older cousins, sisters and aunts as a source of information about sexuality and childbearing near the time of their marriage [53]. For some, television adverts were the main source of information on contraception, although they lacked full understanding of how to use and access these [51].

#### Restrictive gender norms limit young women’s sexual and reproductive decision making

The importance of gender norms was described in all included articles. Nine articles emphasized how underlying gender norms in South Asia meant that young women were often unable to exercise sexual consent and lacked control over their own reproductive health [44,46,50,53–56,58,59]. These articles primarily discussed three broad sub-themes stemming from the ideal of “good virtue”: gender narratives and a lack of sexual consent for girls and women; gendered mobility restrictions and limited access to sexual health information; and lack of decision making around contraception and its consequences.

**Gender narratives and consent:** Four articles referred to submissive/vulnerable femininity and heroic/hegemonic masculinity as dominant gender narratives in South Asia which contribute towards undermining women’s sexual consent [44,54,55,58]. For example, many boys in a Nepal-based study believed that most girls were “very soft and weakhearted in nature and cannot express their feelings of love” [58]. An unmarried FGD respondent from the same article further elaborated that young men also expected submissiveness in terms of making the first move regarding dating: “In most cases, boys act first. They always push for it [sex]” [58].

According to authors of a 2019 study in Bangladesh, some young men observed pornography as ‘brutally’ reinforcing sexual submissiveness of women. A 23-year-old participant pointed out that, “A boy is always a hero in pornography, a female is subordinate. A woman is treated like an animal” [44].

Similarly, Iyer’s [55] article about young people’s understandings of gender sexual violence identified Bollywood films as a source for dominant discourses in gender. The combination of fighting and pursuing heterosexual romance reinforced both narratives of heroic masculinity as well as vulnerable femininity. In such cases, the author asserts, girls were inevitably cast as passive and helpless, with boys fighting to determine who will “win” her hand. The article further revealed that in the aftermath of Delhi gang rape case, vulnerable femininity held influence at home and at school through the emphasis of being alert in public spaces. As a consequence of these dominant gender discourses, boys often conflated ideas of respecting and protecting girls, thereby undermining the latter’s agency [44,55]. There was also confusion over what constituted legitimate sexual attraction as opposed to predatory sexual behaviour as boys attempted to distinguish themselves from male predator stereotypes. Within a context where sex was frequently discussed in terms of sexual violence, many boys struggled to conceptualize sexual desire in positive terms [55].

Findings from the above study also suggested that “girls’ expectations of greater freedoms can lead them to vociferously challenge attempts at restriction” [55]. One way girls in this study challenged the narrative of vulnerable femininity was by aspiring to independent “can-do” narratives of girlhood–high-achieving, independent femininity–in India:

I think being self-dependent is the most important thing as a girl. If I get married, I don’t want to get married without working in any office or–because I–don’t completely want to depend on my husband, and on my family.

- Girl (age not reported), India, FGD [55]

**Restricted mobility:** Six articles highlighted mobility restrictions–imposed by parents or husbands/in-laws–as one of the main constraints in everyday life of both married and unmarried young women. Public space was identified primarily from a gendered viewpoint as young women’s access to public space was restricted due to safety concerns and fear of harassment and abuse from men [46,55]. Respondents explained that despite changing attitudes towards women in India, concern over safety still led to parents placing restrictions on their daughters [55].

As well as safety concerns–which were reported across all four countries–women’s presence in public spaces was also seen as departing from the ideals of “good virtue” and, therefore, raised suspicion about sexual chastity [46,55]. A 19-year-old respondent from Pakistan gave the example of being afraid that mistrust from her future spouse could lead to restrictions after marriage and suggested that she was “afraid of what will happen after my marriage” [53].

Overall, young women felt frustration, fear, and anger at having to abide by these restrictions [46]. In terms of consequences, restricting girls’ movement in public spaces contributes to school dropout and early marriage, and negatively affects girls’ health and wellbeing [46]. Moreover, Hamid et al. [53] argued that unlike their male counterparts, young women had limited access to different types of media because of their restricted mobility and fewer opportunities from which to choose [53].

Despite this, findings from included articles also demonstrated that young women–of different ages, and educational and socioeconomic backgrounds–challenged mobility restrictions in different ways. For example, most urban participants in a Nepali study about dating and sex mentioned having some dating experience and accessing places such as restaurants, inns, hotels, cinemas, parks, and public transport on dates [58]. While there appeared to be gender segregation in the home–with opposite-sex peers often discouraged from visiting each other’s homes–participants said it was commonplace for young people to form romantic partnerships through school or in their community [58]. For instance, a young woman was able to meet her boyfriend because of frequent opportunities to meet at his shop: “I used to get food and other items from his store…later on we became closer and started to love each other” [58].

**Gendered reproductive health decision making:** Six articles which included experiences of married or engaged participants from urban areas–particularly slums–in India, Nepal, and Pakistan and touched on young women’s lack of control over their own reproductive health [46,50,51,53,56,59]. Husbands and mothers-in-law appeared to be the main decision makers in contraceptive use and childbearing, with the majority of women across the four studies saying that condom use was dependent on their husbands. Conversely, young married men from a study in Karachi, Pakistan stated that the prime responsibility of avoiding unintended pregnancies lay with their partners, although the men themselves also generally avoided using contraception [56]. Young women identified unequal gender dynamics and hierarchy within the marital household as underlying reasons why they could not exercise autonomy over family planning [51,56,59].

As mentioned earlier, Hamid and co-authors explored this lack of reproductive autonomy by interviewing engaged women living in slums in Islamabad, Pakistan. Their findings showed how young women were underprepared for marital life with very little access to sexual health information due to a culture of silence around sexuality and parental pressures to be obedient daughters. The authors concluded that this “socialisation into submissiveness” contributed to women’s lack of control over future reproductive health. Young women in this study reportedly abided by rules and trusted that family support would ensure security in future life. Unlike young people in other studies, these women did not seem to have access to resources outside of parent-approved sources such as older cousins, sisters, and aunts. As well as directing questions about sexuality and childbearing to these sources, young women also felt that television–in particular, contraceptive adverts–was akin to information from a peer from which they could learn about sexuality. Bankar et al. [46] and Sekine el al. [59] also found that young women’s restricted access to public space and resources persisted after marriage.

In their study of pregnant women aged 15 to 24 in Kathmandu, Nepal, Deuba et al. [50] identified a lack of women’s sexual consent and strong son preference among slum residents as contributing factors of prepartum intimate partner violence among participants. These young pregnant women were more likely to experience different forms of violence (psychological, physical, and sexual) if they refused to have sex with their husband, gave birth to a girl, or if their husband had alcohol use disorder. One of the main misconceptions around son preference was that having sex during pregnancy would result in a son. This misconception, coupled with a lack of women’s sexual consent, often led to experiences of sexual violence [50]. Additionally, most young women in Deuba et al’s [50] study reported (mis)information around son preference from their spouses and mothers-in-law. Similar misconceptions were also reported by middle-class respondents in Nepal [59].

In terms of coping with IPV, most of these young women reported tolerating and accepting abuse due to economic dependence on their husbands. In some cases, their in-laws interfered to prevent further escalation of physical abuse and sometimes women were able to seek informal support from close family members [50].

#### Heteronormativity presumed and implied in research through absence of sexual and gender diversity

All included research appeared to be about (presumed) cisgender heterosexual and able-bodied individuals, with only two articles mentioning sexual diversity. A study about young men’s experiences of sex education in Bangladesh mentioned the lack of queer representation in their research as a limitation due to difficulties in accessing “a more diverse group of participants representing different sexualities in Bangladesh where such sexual diversity is not acknowledged socially or legally” [44]. A study exploring perceptions of sexuality and sexual health education among college students in Pune, India touched very briefly on the topic of “homosexuality and anal sex”. Authors reported “mixed reactions” on homosexuality from boys in the study–no data were presented on perceptions of girls. Boys from lower socioeconomic backgrounds were said to lack awareness about homosexuality while others described it as a “personal choice”. The findings finally stated that many explored homosexuality as “a different way of fun” although this was not elaborated any further. It was unclear how the authors defined homosexuality in this case or how the respondents themselves identified as this was not reported [48].

While participants in Khan and Raby’s study all self-identified as heterosexual, other articles did not explicitly report respondents’ sexual or gender identity. At the same time, the included articles did not present any findings specific to experiences of non-heterosexual and non-cisgender young people. Therefore, it was not within the scope of these included articles to explore, and provide recommendations, around sexual health implications of different sexual or gender identities. Findings from articles looking at sexual health needs and barriers, for instance, focussed on overall issues such as concerns around privacy and confidentiality of services, accessibility of services, and lack of sexual health knowledge [45,51,52,57].

The use of particular definitions also excluded young people who may not identify as heterosexual or cisgender. For example, a paper on perceptions around dating and sex in Nepal defined dating as “a meeting between *young women and men* for romantic and sexual purposes” without indicating that the study was concentrating only on heterosexual relationships [58]. Similarly, Iyer’s mixed methods study of middle-class students in India reflected heteronormativity as default by framing heterosocial (cross-sex) friendships as having potential for romance–and therefore being discouraged by educational institutions–while homosocial (same-sex) friendships were subsequently seen as only platonic. A study based on the puberty experiences of men in Pakistan also only referred to “societal norms encouraged them to exhibit opposite-sex attraction” without addressing the respondents’ sexual identities further [45].

## Discussion

This review shows how parental expectations of premarital sexual abstinence and silence around sexuality contributed to inadequate sexual health information for young people, and restrictive gendered norms (such as dominant gender narratives and mobility restrictions) limited young women’s sexual and reproductive decision making. None of the studies addressed diversity of sexuality or gender identity.

Findings around a lack of intergenerational sexuality communication between young people and older adults are extensively supported by literature reporting on silence around sexuality [8,26,45,47,60–63]. As with other global studies, our review found that lack of communication was predominantly reported as being due to embarrassment or shame around the topic of sexuality as well as expectations of premarital sexual abstinence [60,61,63]. While the included articles focused on parental norms towards unmarried young people, perceived norms among young people could also have hindered obtaining necessary information on sexual health. For example, young people had hesitations in talking about sexuality with their parents. Although not all young people had access to alternative sources of sexuality information, most participants in our included studies navigated the gap in sexuality communication by looking for information via the internet, media, pornography, and their peer groups [44,48,49,51,58].

Again, this finding is supported by wider literature [60,61,64]. For example, while peer relationships were considered a valuable–although at times unreliable–source of sexual learning, there is mixed evidence on the effectiveness of peer education contributing to SRH knowledge, attitudes and behaviour [44,48,51,54,57,65].

The included studies showed how gender had an impact on experiences of all relationships, particularly in terms of cross-sex communications around sexuality and in terms of gender segregation. Son preference, gender inequality, early marriage and its impact on young people in South Asia has been well documented [24]. Our review found that parents and educational institutions encouraged gender segregation at school as a way of ensuring premarital “sexual purity” [44,49,54]. Restrictive gendered norms, such as dominant gender narratives around vulnerable/submissive femininity and hegemonic/heroic masculinity, particularly affected young women’s sexual and reproductive decision making. Puberty has been found to be a time for expanded participation in public life for boys and intensifying restrictions for girls in South Asia [66–68], and there are high levels of institutional and societal gender discrimination across all four countries included in our review [69].

Included studies engaged with expectations of premarital “sexual purity”, silence around sexuality, and restrictive gender norms as experienced by both married and unmarried young people from different socioeconomic and educational backgrounds. However, there were noticeable gaps in terms of reporting on particular social orientations, such as young people with disabilities or gender and sexual diverse individuals. Does this lack of reporting imply the authors presumed all their interviewees were cisgender and heterosexual? This gap in reporting has implications for our understanding of non-heterosexual and non-cisgender young people’s sexual health within heteronormative societies–with the view that institutionalized heterosexuality constitutes “the standard for legitimate and expected social and sexual relations” [70]. We observed that the absence of more diverse narratives meant that the included articles could not provide recommendations that reflected the specific sexual health needs of these heterogenous communities–such as accessibility of sexual health services, contraceptive use, communication and education around sexuality, and experiences of harassment and violence. Could this erasure from research mean that diverse lived experiences and health needs continue to be unaddressed in mainstream research or policy? Without such research, it is difficult to contextualize, and potentially challenge, the assumption that heteronormative experiences are universal.

There is an urgent need for research examining lived experiences of, and health inequalities within, sexual and gender diverse communities [19,71–74]. We also need global research about “the role of heteronormativity in healthcare and the application of diversity-affirming care into healthcare practices” [19]. Heteronormative patriarchy continues to be a dominant paradigm in South Asia [44,71,75–78]. For example, an article by one of the authors of two Nepal-based studies included in our review emphasized the “lack of understanding of health and well-being, social exclusion, stigma, and discrimination” as experienced by LGBT+ populations in Nepal [71]. Research focussing on diverse sexual and gender identities could shed light on current challenges faced by sexual and gender minoritized (SGM) individuals as well as culturally sensitive ways of navigating discrimination against SGM young people in healthcare and policy.

Five of the included articles exploring young people’s experiences of marriage as an important life event were based on data from urban slums in India, Pakistan, and Nepal [46,47,50,53,56]. Three of these looked specifically at experiences of young women and all focussed on either contraceptive use, intimate partner violence, or mobility restrictions. One reason that the included articles selected slum residents could be to explore intersections of poverty and reproductive health concerns associated with early marriage. Poorer women are statistically more likely to be married during childhood than their richer counterparts in South Asia and are therefore faced with more health consequences associated with early marriage [79].

While a number of articles included married young people, it is plausible that the findings from these studies may also apply to some extent to “other young women from similar backgrounds and in similar situations” [53]. In fact, wider literature confirms that married South Asian women living in slums are confronted with IPV, a lack of decision making around reproductive health, and son preference [80–85]. At the same time, there is a lack of exploratory research where young married people raise their own health needs beyond contraceptive use, childbearing, and gender-based violence. This could reflect a global development agenda to delay early marriage and pregnancy and promote access to, and use of, contraception [66,79]. While these are certainly relevant avenues of inquiry given the high rate of early marriage and widespread gender inequality, the limited body of work on reproductive behaviour of married young people also leaves a gap in narratives about sexual behaviour and pleasure.

Overall, the majority of included articles focussed on young people’s perceptions and attitudes around sexuality, rather than a fuller exploration of lived experiences. While offering valuable insight into sexuality norms and young people’s understanding of sexuality, most studies did not fully explore lived experiences or meanings of sexuality. There was a lack of “thick description” [86] in the articles, and little exploration of participant perspectives on meanings of sexuality, sexual health, sexual wellbeing, and illness. For instance, urban college students in Pune suggested that they wanted parental involvement and discussions on sexuality to obtain knowledge and guidance [48]. However, the research did not probe further or provide in-depth details of how this would happen from an everyday standpoint given the taboo and embarrassment around discussing sexuality as well as norms of premarital purity as reported in most included articles.

Three articles were based on ethnographic research which provided more in-depth detail about the social context on silence around sexuality, and gender norms and sexual violence [49,54,55]. More work like this is needed to “capture the full range of influences on sexual behaviour” [26]. Researchers must acknowledge heterogeneity of experiences and select respondents whose lived experiences may further our understanding of sexuality. For instance, Farid-ul-Hasnain et al. [51] reported on perceptions of HIV/AIDS and safe sex but did not include lived experiences of people living with HIV. This stops the conversation at “people living with HIV are stigmatized” but not how this plays out in the lifeworld of young people living with HIV and how they can be better supported.

Our study had several limitations. Firstly, we used broader sexual health terms rather than searching for specific sexual health issues–e.g. menstruation, consequence of breaking with social norms, or consequences of non-sanctioned sexual behavior such as unwanted pregnancy, abortion, etc. Secondly, we did not include book chapters in the search, although these could contain peer-reviewed empirical findings. Thirdly, our inclusion criteria meant that we did not review studies exploring sexual health issues within an adult population. Similarly, it was also outside the scope of this review to explore quantitative research as well as studies conducted in other South Asian countries or rural settings. Finally, most of the included qualitative data were gathered in another language and then translated into English, implying a certain distance that the quotes have from the actual respondents’ words.

Given the dearth of published qualitative studies on sexual and reproductive health of young people in low- and middle-income countries, the opportunities for future research are plenty [1,3,4]. Qualitative research can provide much-needed in-depth insight into how young people give meaning to, and experience, their sexual health. The inclusion of young people with diverse lived experiences–such as those who identify as LGBTQIA+, youth living with HIV or disabilities etc–would help to address the high unmet SRH needs of “key populations” [4]. Gaps also remain in what we understand about young people’s sexual health throughout South Asia in terms of heterogeneities such as religious differences, refugee populations and so on. By making the research process as participatory and inclusive as possible, young people can set their own health priorities and inform policy through sharing their lived experiences. Health needs and experiences are not static and vary across socioeconomic situations, gender and sexual identities, marital status, and stages of life–the sexual health needs and lived experiences of a married young man may be different to his needs and experiences as a young adolescent boy going through puberty, for example. Thus, it is important to continue efforts to fill research gaps and increase our understanding of these differentials within the local context of socio-sexual and gender norms. Asking more detailed questions and collecting more detailed information about social context through ethnographic health research may be amenable to capturing nuanced experiences on a continuum. Exploratory in-depth research can also be used to consider multiple perspectives, such as young people who do not conform to heteronormativity or able-bodied narratives, to reveal broader and more inclusive contextual meanings of sexuality.

## Supporting information

S1 PRISMA Checklist PRISMA 2020 checklist. (DOCX)

S1 Table Search strategies for electronic databases. (DOCX)

S2 Table List of inclusion-exclusion criteria. (DOCX)

S3 Table Results for Critical Appraisal Skills Programme (CASP) Qualitative Checklist. (DOCX)

S1 Text Coding framework for data extraction. (DOCX)
