# Supplementary material for: Socio-sexual norms and young people’s sexual health in urban Bangladesh, India, Nepal and Pakistan: A qualitative scoping review
Source: PLOS Glob Public Health. 2024 Feb 20;4(2):e0002179. doi: 10.1371/journal.pgph.0002179 (PMC10878529; doi:10.1371/journal.pgph.0002179)
Supplement: S1 Table — (DOCX) [file pgph.0002179.s002.docx]

Search strategies for electronic databases

*Search terms for Ovid (Embase, Medline, PyscInfo, Global Health)*

| 1. (youth or young people or young person or young adult* or young male* or young female* or adolesc* or teen* or girl or boy or girls or boys).ti,ab. |
| --- |
| 2.exp adolescent/ |
| 3. 1 or 2 |
| 4. (sexua* or sexual health or sexual behavio?r).ti,ab. |
| 5. (reproduc* or reproductive health or reproductive behavio?r).ti,ab. |
| 6. (SRH or SRHR).ti,ab. |
| 7. (sexual or reproductive) adj3 (health or behavio?r or rights).ti,ab. |
| 8. exp sexual health/ or exp reproductive health/ or exp sexual behavior/ or exp reproductive behavior/ or exp sexuality/ |
| 9. 4 or 5 or 6 or 7 or 8 |
| 10. (Bangladesh or India or Pakistan or Nepal or Sout* Asia).ti,ab. |
| 11. exp Bangladesh/ or exp India/ or exp Pakistan/ or exp Nepal/ |
| 12. 10 or 11 |
| 13. (qualitative or ethnograph* or phenomenol* or grounded theor* or experience* or narrative* or ethnolog* or focus group* or interview*).ti,ab. |
| 14. exp qualitative research/ |
| 15. 13 or 14 |
| 16. 3 and 9 and 12 and 15 |

*APA PsycInfo: The subject heading 'adolescent' and ‘Bangladesh’ invalid, Global Health: The subject heading 'sexual behavior' and 'qualitative research' invalid*

*Search terms for PubMed*

| 1. youth[Title/Abstract] OR young people[Title/Abstract] OR young person[Title/Abstract] OR young adult*[Title/Abstract] OR young male*[Title/Abstract] OR young female*[Title/Abstract] OR adolesc*[Title/Abstract] OR teen*[Title/Abstract] OR girl[Title/Abstract] OR boy[Title/Abstract] OR girls[Title/Abstract] OR boys[Title/Abstract] |
| --- |
| 2. adolescent[MeSH Terms] |
| 3. 1 OR 2 |
| 4. sexua*[Title/Abstract] OR "sexual health"[Title/Abstract] OR sexual behavio* [Title/Abstract] |
| 5. reproduc*[Title/Abstract] OR "reproductive health"[Title/Abstract] OR reproductive behavio*[Title/Abstract] |
| 6. SRH[Title/Abstract] OR SRHR[Title/Abstract] |
| 7. sexuality[MeSH Terms] OR sexual behavior[MeSH Terms] OR reproductive health[MeSH Terms] OR reproductive behavior[MeSH Terms] |
| 8. 4 OR 5 OR 6 OR 7 |
| 9. Bangladesh[Title/Abstract] OR India[Title/Abstract] OR Pakistan[Title/Abstract] OR Nepal[Title/Abstract] OR Sout* Asia[Title/Abstract] |
| 10. Bangladesh[MeSH Terms] OR India[MeSH Terms] OR Pakistan[MeSH Terms] or Nepal[MeSH Terms] |
| 11. 9 OR 10 |
| 12. qualitative[Title/Abstract] OR ethnograph*[Title/Abstract] OR phenomenol*[Title/Abstract] OR grounded theor*[Title/Abstract] OR experience*[Title/Abstract] OR narrative*[Title/Abstract] OR ethnolog*[Title/Abstract] OR focus group*[Title/Abstract] OR interview*[Title/Abstract] |
| 13. qualitative research[MeSH Terms] |
| 14. 12 OR 13 |
| 15. 3 AND 8 AND 11 AND 14 |
