# Supplementary material for: Socio-sexual norms and young people’s sexual health in urban Bangladesh, India, Nepal and Pakistan: A qualitative scoping review
Source: PLOS Glob Public Health. 2024 Feb 20;4(2):e0002179. doi: 10.1371/journal.pgph.0002179 (PMC10878529; doi:10.1371/journal.pgph.0002179)
Supplement: S2 Table — (DOCX) [file pgph.0002179.s003.docx]

List of inclusion-exclusion criteria

|  | **Inclusion** | **Exclusion** |
| --- | --- | --- |
|  | Articles published on/after 2010 | Articles published before 2010 |
|  | Peer-reviewed empirical research | Reviews, opinion pieces, book chapters, papers that are not peer reviewed |
|  | Qualitative research or mixed method with qualitative analysis (i.e. not only as part of quantitative survey) | Quantitative studies/no qualitative data collection or analysis |
|  | Focussed on experiences of young people with data that can be disaggregated for respondents aged 15-24 years | Studies looking only at adults (>24) or children (<15) or indistinguishable populations (e.g. 15-49 with no disaggregated data for 15-24 year olds) |
|  | Focussed on any experiential aspect of sexuality and sexual health of young people in everyday life settings (e.g. school, university, workplace, public spaces etc) | Health/non-health issues outside of SRHR (e.g. nutrition, smoking) |
|  | Studies based in Bangladesh, India, Pakistan and Nepal | Studies based outside Bangladesh, India, Pakistan or Nepal (e.g. Bangladeshi diaspora in the UK) |
|  | Studies based in urban areas, or urban and rural areas | Studies focussed only on rural areas |
