# Supplementary material for: Socio-sexual norms and young people’s sexual health in urban Bangladesh, India, Nepal and Pakistan: A qualitative scoping review
Source: PLOS Glob Public Health. 2024 Feb 20;4(2):e0002179. doi: 10.1371/journal.pgph.0002179 (PMC10878529; doi:10.1371/journal.pgph.0002179)
Supplement: S1 Text — (DOCX) [file pgph.0002179.s005.docx]

Coding framework for data extraction

***General***

- Title
- Authors
- Year of publication
- Journal
- Country of study
- Abstract
- Funding agency

***Data collection and study design***

- Year of data collection
- Duration of study
- Location of study
- Study setting
- Study population
- Sample size
- Sampling strategy
- Recruitment process
- Study design
- Reviewers’ remarks on study design and sampling

***Study findings***

- Research topic
- Research findings and themes
- Quotes/findings of interest to reviewers
- Interventions
- Theories
- Norms
- Recommendations
- Reviewers’ remarks on findings
